# Supplementary material for: The Effect of Strengthened Physical Education on Academic Achievements in High School Students: A Quasi-Experiment in China
Source: Int J Environ Res Public Health. 2019 Nov 25;16(23):4688. doi: 10.3390/ijerph16234688 (PMC6926946; doi:10.3390/ijerph16234688)
Supplement: Supplementary file 1 [file ijerph-16-04688-s001.pdf]

Table S1. Associations of Strengthened Physical Education with Academic Performance among Group Sports and Individual Sports in Two and Three Semester Follow-up.

		Coefficient	95% CI	*p*-value	Sample Size
Panel A. Effect on Change in Scores By Sports Type Over Two Semesters					
Group Sports					
	Chinese	0.61	[0.48,0.74]	<0.001	310
	English	0.26	[0.12,0.40]	0.02	310
	Maths	−0.18	[−0.32,−0.04]	<0.001	310
Individual Sports					
	Chinese	0.59	[0.31,0.87]	<0.001	302
	English	0.26	[0.12,0.39]	<0.001	302
	Maths	−0.37	[−0.56,−0.19]	<0.001	302
Panel B. Effect on Change in Scores Over Three Semesters					
		**Coefficient**	**95% CI**	***p*-value**	**Sample Size**
Group Sports					
	Chinese	0.24	[0.01,0.47]	0.05	290
	English	−0.13	[−0.32,0.07]	0.18	290
	Maths	−0.20	[−0.37,−0.03]	0.02	290
Individual Sports					
	Chinese	0.27	[−0.01,0.54]	0.06	282
	English	−0.25	[−0.47,−0.03]	0.03	282
	Maths	−0.26	[−0.43,−0.09]	0.01	282

Notes: Group sports include football, volleyball, and aerobics.
Individual sports include badminton, table tennis and tennis.
